# Crystal structure and DFT study of the zwitterionic form of 3-{(*E*)-1-[(4-ethoxyphenyl)iminiumyl]ethyl}-6-methyl-2-oxo-2*H*-pyran-4-olate

**DOI:** 10.1107/S2056989018000919

**Published:** 2018-01-16

**Authors:** Amel Djedouani, Barkahem Anak, Salima Tabti, Franck Cleymand, Michel François, Solenne Fleutot

**Affiliations:** a Laboratoire de Physicochimie Analytique et Cristallochimie de Matériaux Organométalliques et Biomoléculaires, Université de Constantine 1, 25000 Constantine, Algeria; b Ecole Normale Supérieure de Constantine Assia Djebbar, Ville Universitaire Ali Mendjeli, Constantine 25000, Algeria; c Laboratoire de Chimie des Matériaux, Université de Constantine 1, 25000 Constantine, Algeria; d Université Bachire El Ibrahimi de Bordj Bou Arraridj, Algeria; e Institut Jean Lamour UMR 7198, Parc de Saurupt, CS 14234 F 54042 Nancy, France

**Keywords:** crystal structure, Schiff base, zwitterion, hydrogen bonding, C—H⋯π inter­actions, π–π inter­actions

## Abstract

The title Schiff base compound, C_16_H_17_NO_4_, crystallizes as a zwitterion, with the phenolic H atom having been transferred to the imino group. The resulting iminium and hy­droxy groups are linked by an intra­molecular N—H⋯O hydrogen bond, enclosing an *S*(6) ring motif. The conformation about the C=N bond is *E* and the dihedral angle between the benzene and pyran rings is 70.49 (6)°. In the crystal, mol­ecules are linked by C—H⋯O hydrogen bonds, forming a three-dimensional supra­molecular structure. There are also C—H⋯π inter­actions and offset π–π inter­actions, involving the pyran rings [inter­centroid distance = 3.4156 (8) Å], which consolidate the three-dimensional structure. Quantum chemical calculations of the mol­ecule are in good agreement with the solid state keto–amine (NH) form of the title compound.

## Abstract 2

The title Schiff base compound crystallizes in the zwitterionic form. The resulting iminium and hydroxyl groups are linked by a charge-assisted intra­molecular N^+^—H⋯O^−^ hydrogen bond, and the conformation about the C=N bond is *E*.

## Chemical context   

Hy­droxy Schiff bases have been studied extensively for their biological, photochromic and thermochromic properties (Garnovskii *et al.*, 1993 ▸; Hadjoudis *et al.*, 2004 ▸). They can be used as potential materials for optical memory and switch devices (Zhao *et al.*, 2007 ▸). Proton transfer in these compounds forms the basis for an explanation of the mechanisms of various biological processes where proton transfer is the rate-determining step (Lussier *et al.*, 1987 ▸). In general, *O*-hy­droxy Schiff bases exhibit two possible tautomeric forms, the phenol–imine (or benzenoid) and keto–amine (or quinoid) forms. Depending on the tautomers, two types of intra­molecular hydrogen bonds are possible: O—H⋯N in benzenoid and N—H⋯O in quinoid tautomers. *O*-hy­droxy Schiff bases have been observed in the keto form, in the enol form or in an enol/keto mixture (Nazır *et al.*, 2000 ▸; Antonov *et al.*, 2000 ▸) due to the H-atom transfer. Another form of the Schiff base compounds is their zwitterionic form (Ogawa & Harada, 2003 ▸). Zwitterions of Schiff bases have an ionic intra­molecular hydrogen bond (N^+^—H⋯O^−^) and their N^+^—H bond lengths are longer than the normal bond length observed for neutral N—H bonds (0.87 Å). The mol­ecular structure of the title compound is similar to that of (*E*)-4-hy­droxy-3-[*N*-(4-hy­droxy­phen­yl)ethanimido­yl]-6-methyl-2*H*-pyran-2-one (Djedouani *et al.*, 2015 ▸), which also crystallizes as a zwitterion.
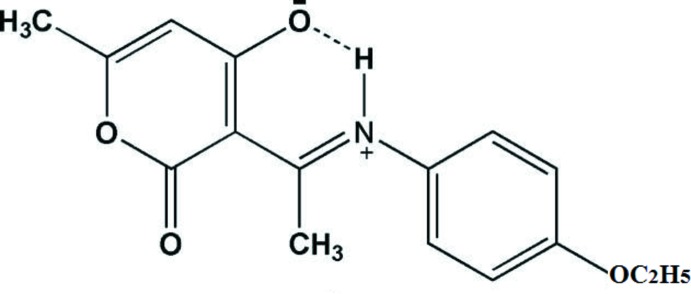


## Structural commentary   

**Figure 1:**
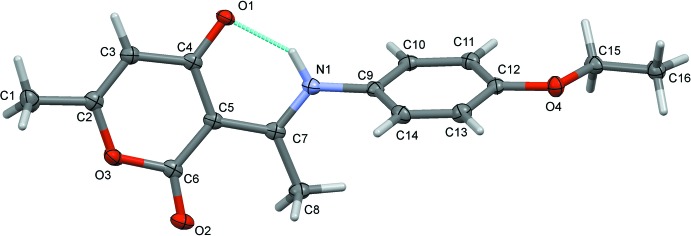
A view of the mol­ecular structure of the title compound with the atom labelling. Displacement ellipsoids are drawn at the 50% probability level, and the intra­molecular N—H⋯O hydrogen bond (see Table 1 ▸) is shown as a dashed line.

The mol­ecular structure of title compound is shown in Fig. 1 ▸. It crystallizes in the zwitterionic form, with the phenolic H atom having been transferred to the imino group. The H atom, H1*N*, was located in a difference-Fourier map and freely refined (N—H = 0.90 (2) Å). The resulting iminium and hy­droxy groups are linked by an intra­molecular N—H⋯O hydrogen bond forming an *S*(6) loop (Fig. 1 ▸ and Table 1 ▸). The dihedral angle between the benzene (C9–C14) and pyran (O3/C2–C6) rings is 70.49 (6)°. The carbon–nitro­gen bond N1=C7 is 1.318 (2) Å, which agrees with values observed in related compounds (Girija & Begum, 2004 ▸; Girija *et al.*, 2004 ▸). It is slightly longer than a typical C=N bond [1.283 (4) Å; Bai & Jing, 2007 ▸], but much shorter than a C—N bond. The N1—C9 bond length is 1.436 (2) Å because of resonance. The carbon–carbon bond connecting the enol and imine groups exhibits inter­mediate distances between those of single and double bond, but being closer to the latter; C5—C7 = 1.427 (2) and C5—C6 = 1.443 (2) Å, reflecting the zwitterionic character of the title compound (Wojciechowski *et al.*, 2003 ▸). The C4—O1 bond length [1.259 (2) Å] is inter­mediate between single and double carbon-to-oxygen bond lengths (1.362 and 1.222 Å, respectively), whereas C6—O2 is 1.215 (2) Å.

The aromatic ring and de­hydro­acetic acid ring are in a *trans* position with respect to the C7=N1 bond, the dihedral angle between the two rings is 70.46 (9)° and the mol­ecular conformation is determined by the presence of the intra­molecular N^+^—H⋯O^−^ hydrogen bond (Fig. 1 ▸ and Table 1 ▸), which generates an *S*(6) ring motif. Similar intra­molecular hydrogen bonds have been reported in other zwitterionic phenolates (Huang *et al.*, 2006 ▸; Temel *et al.*, 2006 ▸).

## Supra­molecular features   

**Figure 2:**
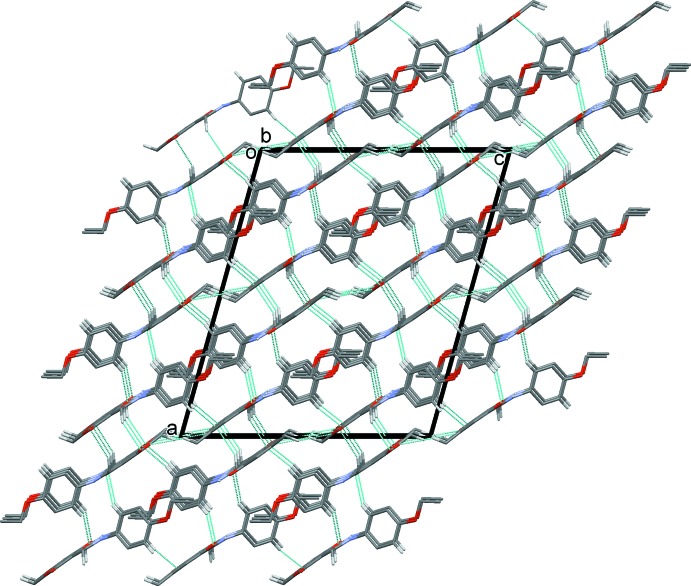
A view along the *b* axis of the crystal packing of the title compound. The hydrogen bonds are shown as dashed lines (see Table 1 ▸), and only the H atoms involved in hydrogen bonding have been included.

In the crystal, mol­ecules are linked by C—H⋯O hydrogen bonds, forming a three-dimensional supra­molecular structure (Fig. 2 ▸ and Table 1 ▸), which is consolidated by C—H⋯π inter­actions (Table 1 ▸) and offset π–π inter­actions. The latter involve symmetry-related pyran rings with a *Cg*⋯*Cg* ^i^ distance of 3.416 (1) Å [*Cg* is the centroid of ring O3/C2–C6, inter­planar distance = 3.319 (1) Å, offset = 0.81 Å, symmetry code (i): −*x* + 1, *y*, −*z* + 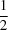].

## Database survey   

A search of the Cambridge Structural Database (Version 5.38, update May 2017; Groom *et al.*, 2016 ▸) for similar structures revealed the presence of three zwitterionic compounds of inter­est, namely (*E*)-6- methyl-2-oxo-3-[1-(*p*-tolyl­iminio)eth­yl]-2*H*-pyran-4-olate (REZMAL; Djedouani *et al.*, 2007 ▸) and 6-methyl-2-oxo-3-[1-(ureidoiminio)eth­yl]-2*H*-pyran-4-olate monohydrate (HOFPOI; Djedouani *et al.*, 2008 ▸) and (*E*)-4-hy­droxy-3-[*N*-(4-hy­droxy­phen­yl)ethanimido­yl]-6-methyl-2*H*-pyran-2-one (CUGPAX; Djedouani *et al.*, 2015 ▸). The mol­ecular conformations of all three compounds are also determined by the presence of an intra­molecular charge-assisted N^+^—H⋯O^−^ hydrogen bond (see Fig. 1 ▸ and Table 1 ▸ for the title compound), which generates an *S*(6) ring motif. Two of these compounds, REZMAL and CUGPAX, have a benzene ring inclined to the pyran ring by 42.25 (10) and 53.31 (11)°, respectively. This is significantly different from the equivalent dihedral angle of 70.46 (9)° in the title compound, which has five hydrogen bonds, two from the eth­oxy group in the *para* position of the benzene and another from the benzene ring, which has increased the dihedral angle between the two rings. On the other hand, CUGPAX has three hydrogen bonds and only one single bond of the hy­droxy group in the *para* position of benzene ring, and the dihedral angle between the two rings is 53.31 (11)°. REZMAL shows only two hydrogen bonds, neither of which involve benzene ring, and the dihedral angle is 42.25 (10)°.

## Density functional study – geometry optimization and mol­ecular orbital calculations   

Geometry optimization and mol­ecular orbital calculations were carried out with the Guassian*09* software package (Frisch *et al.*, 2009 ▸) and the Gaussview visualization program (Dennington *et al.*, 2007 ▸; Rassolov *et al.*, 1998 ▸), using the three-parameter hybrid function of Becke based on the correlation function (B3LYP) of Lee *et al.* (1998 ▸) and Miehlich *et al.* (1989 ▸), with the 6-311G, 6-311G(+) and 6-311G(++) basis sets. The bond lengths, bond angles corresponding to the optimized geometry obtained using the DFT/B3LY P method are given in Table 2 ▸. The calculated C4—C5 bond distance is 1.447 Å correlates nicely with experimental value. The calculated bond lengths with B3LYP/6-311G(++) level are slightly shorter than the experimental values within 0.004–0.035 Å. The calculated bond angles C5—C4—O4 and C4—C5—C7 are close to 120° since atoms C4 and C5 have sp^2^ hybridization. In general, the calculated values are in good agreement with the experimental data.

**Figure 3:**
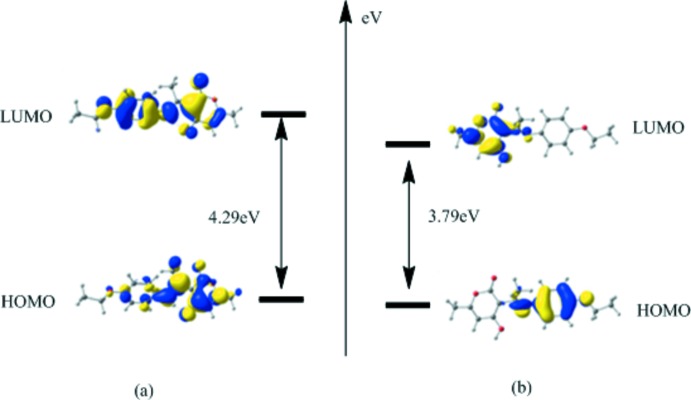
The frontier mol­ecular orbitals for the two possible tautomeric forms, the keto–amine (NH) and the phenol–imine (OH) forms, of the title Schiff base compound.

The highest occupied mol­ecular orbitals (HOMO) and lowest unoccupied orbitals (LUMO) are named frontier orbitals (FMOs). The calculated values at the B3LYP/6-311G(++) level are presented in Table 3 ▸, and the nature of the frontier mol­ecular orbitals for the two possible tautomeric forms, the keto–amine (NH) and the phenol–imine (OH) forms of zwitterionic forms of Schiff bases, are plotted in Fig. 3 ▸. The band-gap energy values calculated for keto–amine (NH) forms were found to be 4.297 eV, which is a large HOMO–LUMO energy gap, implying a higher mol­ecular stability than for the phenol–imine (OH) form, which has a smaller energy gap with the difference between the HOMO and LUMO being 3.791 eV. The HOMO–LUMO energy gap is very important for the chemical activity and explains the eventual charge-transfer inter­action within the mol­ecule. Clearly, the larger HOMO–LUMO gap calculated for the keto–amine (NH) form is in agreement with the stability of the mol­ecule in the solid state.

## Synthesis and crystallization   

The title compound was prepared according to a literature method (Djedouani *et al.*, 2007 ▸). Colourless plate-like crystals were obtained by slow evaporation of a solution in ethanol.

## Refinement   

Crystal data, data collection and structure refinement details are summarized in Table 4 ▸. The NH H atom was located in a difference-Fourier map and freely refined. The C-bound H atoms were included in calculated positions and treated as riding: C—H = 0.95–0.99 Å, with *U*_iso_(H) = 1.5*U*_eq_(C-meth­yl) and 1.2*U*_eq_(C) for other H atoms.

## Supplementary Material

Crystal structure: contains datablock(s) I, _Global. DOI: 10.1107/S2056989018000919/ex2003sup1.cif

Structure factors: contains datablock(s) I. DOI: 10.1107/S2056989018000919/ex2003Isup2.hkl

Supporting information file. DOI: 10.1107/S2056989018000919/ex2003Isup3.cml

CCDC reference: 1816916

Additional supporting information:  crystallographic information; 3D view; checkCIF report

## Figures and Tables

**Table 1:** Hydrogen-bond geometry (Å, °) *Cg*1 is the centroid of the C9–C14 benzene ring.

*D*—H⋯*A*	*D*—H	H⋯*A*	*D*⋯*A*	*D*—H⋯*A*
N1—H1*N*⋯O1	0.90 (2)	1.74 (2)	2.5411 (15)	147 (2)
C1—H1*B*⋯O3^i^	0.98	2.57	3.4461 (18)	149
C8—H8*B*⋯O2^ii^	0.98	2.62	3.3478 (17)	132
C10—H10⋯O1^iii^	0.95	2.56	3.2035 (16)	125
C13—H13⋯O2^iv^	0.95	2.46	3.3950 (17)	170
C15—H15*A*⋯*Cg*1^v^	0.99	2.74	3.618 (2)	149

Symmetry codes: (i) 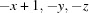; (ii) 
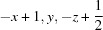
; (iii) 
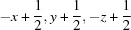
; (iv) 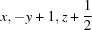; (v) 
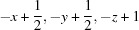
.

**Table 2:** DFT and X-ray geometric parameters (Å, °) for the title compound

	B3LYP/6–311g(++)	X-ray data
N1—C7	1.334	1.318 (2)
C5—C7	1.423	1.427 (2)
C5—C4	1.447	1.447 (2)
C4—O1	1.253	1.259 (2)
C7—C8	1.460	1.495 (2)
		
H1*N*—N1—C7	112.29	112.0 (11)
N1—C7—C5	118.07	118.27 (13)
C4—C5—C7	120.75	120.58 (12)
C5—C4—O4	123.27	123.19 (13)

**Table 3:** Frontier mol­ecular orbital energies (eV): HOMO–LUMO gap of the keto–amine (NH) and phenol–imine (OH) forms of the title compound

Energy	keto–amine (NH) form	phenol–imine (OH) form
E_HOMO_	6.167	5.491
E_LUMO_	1.870	1.700
E_gap_	4.297	3.791

**Table 4:** Experimental details

Crystal data	
Chemical formula	C_16_H_17_NO_4_
*M*_r_	287.30
Crystal system, space group	Monoclinic, *C*2/*c*
Temperature (K)	100
*a*, *b*, *c* (Å)	21.0983 (13), 7.7792 (5), 17.7036 (11)
β (°)	105.564 (2)
*V* (Å^3^)	2799.1 (3)
*Z*	8
Radiation type	Mo *K*α
μ (mm^−1^)	0.10
Crystal size (mm)	0.18 × 0.08 × 0.03

Data collection	
Diffractometer	Bruker APEXII QUAZAR CCD
Absorption correction	Multi-scan (*SADABS*; Bruker, 2004 ▸)
*T*_min_, *T*_max_	0.596, 0.746
No. of measured, independent and observed [*I* > 2σ(*I*)] reflections	17108, 2750, 2315
*R*_int_	0.037
(sin θ/λ)_max_ (Å^−1^)	0.617

Refinement	
*R*[*F* ^2^ > 2σ(*F* ^2^)], *wR*(*F* ^2^), *S*	0.035, 0.094, 1.07
No. of reflections	2750
No. of parameters	197
H-atom treatment	H atoms treated by a mixture of independent and constrained refinement
Δρ_max_, Δρ_min_ (e Å^−3^)	0.23, −0.21

Computer programs: *APEX2* and *SAINT* (Bruker, 2004 ▸), *SHELXS2016/6* (Sheldrick, 2008 ▸), *SHELXL2016/6* (Sheldrick, 2015 ▸), *Mercury* (Macrae *et al.*, 2008 ▸), *PLATON* (Spek, 2009 ▸) and *publCIF* (Westrip, 2010 ▸).

## supplementary crystallographic information

### Crystal data

**Table:**

C_16_H_17_NO_4_	*F*(000) = 1216
*M**_r_* = 287.30	*D*_x_ = 1.364 Mg m^−^^3^
Monoclinic, *C*2/*c*	Mo *K*α radiation, λ = 0.71073 Å
*a* = 21.0983 (13) Å	Cell parameters from 18962 reflections
*b* = 7.7792 (5) Å	θ = 2.0–27.5°
*c* = 17.7036 (11) Å	µ = 0.10 mm^−^^1^
β = 105.564 (2)°	*T* = 100 K
*V* = 2799.1 (3) Å^3^	Plate, colorless
*Z* = 8	0.18 × 0.08 × 0.03 mm

### Data collection

**Table:**

Bruker APEXII QUAZAR CCD diffractometer	2750 independent reflections
Radiation source: ImuS	2315 reflections with *I* > 2σ(*I*)
Graphite monochromator	*R*_int_ = 0.037
f\ and ω scans	θ_max_ = 26.0°, θ_min_ = 2.0°
Absorption correction: multi-scan (SADABS; Bruker, 2004)	*h* = −16→26
*T*_min_ = 0.596, *T*_max_ = 0.746	*k* = −9→9
17108 measured reflections	*l* = −21→21

### Refinement

**Table:**

Refinement on *F*^2^	Primary atom site location: structure-invariant direct methods
Least-squares matrix: full	Secondary atom site location: difference Fourier map
*R*[*F*^2^ > 2σ(*F*^2^)] = 0.035	Hydrogen site location: mixed
*wR*(*F*^2^) = 0.094	H atoms treated by a mixture of independent and constrained refinement
*S* = 1.07	*w* = 1/[σ^2^(*F*_o_^2^) + (0.0419*P*)^2^ + 2.3424*P*] where *P* = (*F*_o_^2^ + 2*F*_c_^2^)/3
2750 reflections	(Δ/σ)_max_ < 0.001
197 parameters	Δρ_max_ = 0.23 e Å^−^^3^
0 restraints	Δρ_min_ = −0.21 e Å^−^^3^

### Special details

**Table:**

Geometry. All esds (except the esd in the dihedral angle between two l.s. planes) are estimated using the full covariance matrix. The cell esds are taken into account individually in the estimation of esds in distances, angles and torsion angles; correlations between esds in cell parameters are only used when they are defined by crystal symmetry. An approximate (isotropic) treatment of cell esds is used for estimating esds involving l.s. planes.

### Fractional atomic coordinates and isotropic or equivalent isotropic displacement parameters (Å^2^)

**Table:**

	*x*	*y*	*z*	*U*_iso_*/*U*_eq_	
O1	0.37595 (5)	−0.09851 (12)	0.25430 (5)	0.0178 (2)	
O2	0.46409 (5)	0.36094 (13)	0.13692 (6)	0.0223 (2)	
O3	0.48126 (5)	0.09177 (13)	0.11188 (5)	0.0203 (2)	
O4	0.28470 (5)	0.53164 (13)	0.53959 (6)	0.0195 (2)	
N1	0.35473 (6)	0.20560 (15)	0.29392 (7)	0.0167 (3)	
H1N	0.3533 (9)	0.090 (2)	0.2929 (10)	0.033 (5)*	
C1	0.51239 (8)	−0.1830 (2)	0.07643 (9)	0.0261 (4)	
H1A	0.558974	−0.151115	0.094154	0.039*	
H1B	0.495675	−0.158824	0.020257	0.039*	
H1C	0.507565	−0.305845	0.085797	0.039*	
C2	0.47443 (7)	−0.08168 (19)	0.12051 (8)	0.0186 (3)	
C3	0.43857 (7)	−0.14438 (18)	0.16567 (8)	0.0179 (3)	
H3	0.433601	−0.265332	0.168807	0.021*	
C4	0.40712 (6)	−0.03355 (17)	0.20989 (8)	0.0154 (3)	
C5	0.41363 (6)	0.14995 (17)	0.20071 (7)	0.0153 (3)	
C6	0.45203 (6)	0.21302 (18)	0.15026 (8)	0.0169 (3)	
C7	0.38639 (6)	0.26807 (18)	0.24515 (8)	0.0162 (3)	
C8	0.39081 (7)	0.45891 (18)	0.23821 (8)	0.0203 (3)	
H8C	0.374124	0.492541	0.183088	0.031*	
H8B	0.436806	0.495022	0.257827	0.031*	
H8A	0.364402	0.514294	0.269172	0.031*	
C9	0.33460 (7)	0.29881 (17)	0.35356 (8)	0.0164 (3)	
C10	0.26863 (7)	0.30913 (17)	0.35148 (8)	0.0171 (3)	
H10	0.236228	0.263353	0.308090	0.020*	
C11	0.24977 (7)	0.38655 (17)	0.41296 (8)	0.0173 (3)	
H11	0.204561	0.393367	0.411788	0.021*	
C12	0.29738 (7)	0.45369 (17)	0.47593 (8)	0.0166 (3)	
C13	0.36385 (7)	0.44554 (18)	0.47719 (8)	0.0193 (3)	
H13	0.396300	0.493437	0.519946	0.023*	
C14	0.38224 (7)	0.36797 (18)	0.41637 (8)	0.0186 (3)	
H14	0.427417	0.361699	0.417316	0.022*	
C15	0.21698 (7)	0.56107 (18)	0.53868 (8)	0.0193 (3)	
H15A	0.192613	0.451001	0.533117	0.023*	
H15B	0.195276	0.637069	0.494438	0.023*	
C16	0.21851 (7)	0.64530 (19)	0.61584 (9)	0.0224 (3)	
H16A	0.245376	0.749760	0.622067	0.034*	
H16B	0.237520	0.565599	0.658818	0.034*	
H16C	0.173613	0.675273	0.616796	0.034*	

### Atomic displacement parameters (Å^2^)

**Table:**

	*U*^11^	*U*^22^	*U*^33^	*U*^12^	*U*^13^	*U*^23^
O1	0.0187 (5)	0.0183 (5)	0.0179 (5)	−0.0034 (4)	0.0075 (4)	0.0023 (4)
O2	0.0232 (5)	0.0233 (5)	0.0203 (5)	−0.0055 (4)	0.0056 (4)	0.0058 (4)
O3	0.0226 (5)	0.0243 (5)	0.0155 (5)	−0.0066 (4)	0.0077 (4)	−0.0002 (4)
O4	0.0167 (5)	0.0222 (5)	0.0200 (5)	0.0015 (4)	0.0058 (4)	−0.0013 (4)
N1	0.0170 (6)	0.0156 (6)	0.0179 (6)	−0.0009 (5)	0.0052 (5)	0.0022 (5)
C1	0.0239 (8)	0.0343 (9)	0.0214 (8)	−0.0039 (7)	0.0083 (6)	−0.0058 (6)
C2	0.0168 (7)	0.0232 (7)	0.0133 (7)	−0.0027 (6)	−0.0003 (6)	−0.0006 (6)
C3	0.0180 (7)	0.0186 (7)	0.0155 (7)	−0.0022 (6)	0.0017 (6)	0.0012 (5)
C4	0.0116 (6)	0.0202 (7)	0.0121 (6)	−0.0029 (5)	−0.0008 (5)	0.0018 (5)
C5	0.0125 (6)	0.0188 (7)	0.0122 (6)	−0.0023 (5)	−0.0008 (5)	0.0024 (5)
C6	0.0139 (7)	0.0232 (7)	0.0108 (6)	−0.0028 (6)	−0.0016 (5)	0.0022 (5)
C7	0.0118 (6)	0.0199 (7)	0.0135 (7)	−0.0020 (5)	−0.0022 (5)	0.0035 (5)
C8	0.0221 (7)	0.0183 (7)	0.0197 (7)	−0.0007 (6)	0.0040 (6)	0.0036 (5)
C9	0.0183 (7)	0.0137 (6)	0.0175 (7)	0.0006 (5)	0.0056 (6)	0.0039 (5)
C10	0.0170 (7)	0.0148 (6)	0.0175 (7)	−0.0012 (5)	0.0012 (6)	0.0031 (5)
C11	0.0146 (7)	0.0164 (7)	0.0209 (7)	0.0020 (5)	0.0045 (6)	0.0038 (5)
C12	0.0199 (7)	0.0141 (6)	0.0166 (7)	0.0022 (5)	0.0063 (6)	0.0026 (5)
C13	0.0170 (7)	0.0197 (7)	0.0198 (7)	−0.0002 (6)	0.0024 (6)	−0.0001 (6)
C14	0.0137 (7)	0.0213 (7)	0.0210 (7)	0.0010 (6)	0.0048 (6)	0.0030 (6)
C15	0.0165 (7)	0.0189 (7)	0.0238 (8)	0.0021 (5)	0.0079 (6)	0.0037 (6)
C16	0.0221 (7)	0.0211 (7)	0.0266 (8)	0.0021 (6)	0.0112 (6)	0.0024 (6)

### Geometric parameters (Å, º)

**Table:**

O1—C4	1.2585 (16)	C8—H8C	0.9800
O2—C6	1.2154 (17)	C8—H8B	0.9800
O3—C2	1.3698 (17)	C8—H8A	0.9800
O3—C6	1.3988 (18)	C9—C10	1.385 (2)
O4—C12	1.3680 (16)	C9—C14	1.392 (2)
O4—C15	1.4427 (16)	C10—C11	1.392 (2)
N1—C7	1.3182 (18)	C10—H10	0.9500
N1—C9	1.4358 (18)	C11—C12	1.387 (2)
N1—H1N	0.902 (19)	C11—H11	0.9500
C1—C2	1.486 (2)	C12—C13	1.398 (2)
C1—H1A	0.9800	C13—C14	1.378 (2)
C1—H1B	0.9800	C13—H13	0.9500
C1—H1C	0.9800	C14—H14	0.9500
C2—C3	1.332 (2)	C15—C16	1.508 (2)
C3—C4	1.440 (2)	C15—H15A	0.9900
C3—H3	0.9500	C15—H15B	0.9900
C4—C5	1.4474 (19)	C16—H16A	0.9800
C5—C7	1.427 (2)	C16—H16B	0.9800
C5—C6	1.4431 (19)	C16—H16C	0.9800
C7—C8	1.4946 (19)		
			
C2—O3—C6	122.46 (11)	H8C—C8—H8A	109.5
C12—O4—C15	118.24 (11)	H8B—C8—H8A	109.5
C7—N1—C9	126.68 (12)	C10—C9—C14	120.28 (13)
C7—N1—H1N	112.0 (11)	C10—C9—N1	120.22 (12)
C9—N1—H1N	120.3 (11)	C14—C9—N1	119.34 (12)
C2—C1—H1A	109.5	C9—C10—C11	120.03 (13)
C2—C1—H1B	109.5	C9—C10—H10	120.0
H1A—C1—H1B	109.5	C11—C10—H10	120.0
C2—C1—H1C	109.5	C12—C11—C10	119.60 (13)
H1A—C1—H1C	109.5	C12—C11—H11	120.2
H1B—C1—H1C	109.5	C10—C11—H11	120.2
C3—C2—O3	121.42 (13)	O4—C12—C11	124.71 (13)
C3—C2—C1	126.45 (14)	O4—C12—C13	115.09 (12)
O3—C2—C1	112.10 (12)	C11—C12—C13	120.19 (13)
C2—C3—C4	121.71 (13)	C14—C13—C12	119.94 (13)
C2—C3—H3	119.1	C14—C13—H13	120.0
C4—C3—H3	119.1	C12—C13—H13	120.0
O1—C4—C3	119.56 (12)	C13—C14—C9	119.95 (13)
O1—C4—C5	123.19 (13)	C13—C14—H14	120.0
C3—C4—C5	117.25 (12)	C9—C14—H14	120.0
C7—C5—C6	119.89 (12)	O4—C15—C16	106.17 (11)
C7—C5—C4	120.58 (12)	O4—C15—H15A	110.5
C6—C5—C4	119.38 (13)	C16—C15—H15A	110.5
O2—C6—O3	113.66 (12)	O4—C15—H15B	110.5
O2—C6—C5	128.60 (14)	C16—C15—H15B	110.5
O3—C6—C5	117.73 (12)	H15A—C15—H15B	108.7
N1—C7—C5	118.27 (13)	C15—C16—H16A	109.5
N1—C7—C8	118.28 (13)	C15—C16—H16B	109.5
C5—C7—C8	123.44 (12)	H16A—C16—H16B	109.5
C7—C8—H8C	109.5	C15—C16—H16C	109.5
C7—C8—H8B	109.5	H16A—C16—H16C	109.5
H8C—C8—H8B	109.5	H16B—C16—H16C	109.5
C7—C8—H8A	109.5		
			
C6—O3—C2—C3	0.80 (19)	C4—C5—C7—N1	−0.68 (18)
C6—O3—C2—C1	−177.59 (11)	C6—C5—C7—C8	4.91 (19)
O3—C2—C3—C4	−2.3 (2)	C4—C5—C7—C8	−179.52 (12)
C1—C2—C3—C4	175.89 (13)	C7—N1—C9—C10	119.67 (15)
C2—C3—C4—O1	−177.10 (12)	C7—N1—C9—C14	−64.93 (18)
C2—C3—C4—C5	2.62 (19)	C14—C9—C10—C11	−1.0 (2)
O1—C4—C5—C7	2.5 (2)	N1—C9—C10—C11	174.36 (12)
C3—C4—C5—C7	−177.19 (12)	C9—C10—C11—C12	0.3 (2)
O1—C4—C5—C6	178.11 (12)	C15—O4—C12—C11	−5.99 (19)
C3—C4—C5—C6	−1.60 (18)	C15—O4—C12—C13	173.59 (12)
C2—O3—C6—O2	178.87 (11)	C10—C11—C12—O4	−179.66 (12)
C2—O3—C6—C5	0.19 (18)	C10—C11—C12—C13	0.8 (2)
C7—C5—C6—O2	−2.5 (2)	O4—C12—C13—C14	179.28 (12)
C4—C5—C6—O2	−178.17 (13)	C11—C12—C13—C14	−1.1 (2)
C7—C5—C6—O3	175.91 (11)	C12—C13—C14—C9	0.4 (2)
C4—C5—C6—O3	0.28 (18)	C10—C9—C14—C13	0.7 (2)
C9—N1—C7—C5	167.68 (12)	N1—C9—C14—C13	−174.74 (12)
C9—N1—C7—C8	−13.4 (2)	C12—O4—C15—C16	179.27 (11)
C6—C5—C7—N1	−176.26 (12)		

### Hydrogen-bond geometry (Å, º)

Cg1 is the centroid of the C9–C14 benzene ring.

**Table:**

*D*—H···*A*	*D*—H	H···*A*	*D*···*A*	*D*—H···*A*
N1—H1*N*···O1	0.90 (2)	1.74 (2)	2.5411 (15)	147 (2)
C1—H1*B*···O3^i^	0.98	2.57	3.4461 (18)	149
C8—H8*B*···O2^ii^	0.98	2.62	3.3478 (17)	132
C10—H10···O1^iii^	0.95	2.56	3.2035 (16)	125
C13—H13···O2^iv^	0.95	2.46	3.3950 (17)	170
C15—H15*A*···*Cg*1^v^	0.99	2.74	3.618 (2)	149

Symmetry codes: (i) −*x*+1, −*y*, −*z*; (ii) −*x*+1, *y*, −*z*+1/2; (iii) −*x*+1/2, *y*+1/2, −*z*+1/2; (iv) *x*, −*y*+1, *z*+1/2; (v) −*x*+1/2, −*y*+1/2, −*z*+1.
